# How can SHAP values help to shape metabolic stability of chemical compounds?

**DOI:** 10.1186/s13321-021-00542-y

**Published:** 2021-09-27

**Authors:** Agnieszka Wojtuch, Rafał Jankowski, Sabina Podlewska

**Affiliations:** 1 Faculty of Mathematics and Computer Science, Jagiellonian University, 6 S. Łojasiewicza Street, 30-348 Kraków, Poland; 2 Maj Institute of Pharmacology, Polish Academy of Sciences, 12 Smętna Street, 31-343 Kraków, Poland; 3 Department of Technology and Biotechnology of Drugs, Faculty of Pharmacy, Jagiellonian University Medical College, 9 Medyczna Street, 30-688 Kraków, Poland

**Keywords:** Metabolic stability, Machine learning, Web service, SHAP, ChEMBL database, Explainability

## Abstract

**Background:**

Computational methods support nowadays each stage of drug design campaigns. They assist not only in the process of identification of new active compounds towards particular biological target, but also help in the evaluation and optimization of their physicochemical and pharmacokinetic properties. Such features are not less important in terms of the possible turn of a compound into a future drug than its desired affinity profile towards considered proteins. In the study, we focus on metabolic stability, which determines the time that the compound can act in the organism and play its role as a drug. Due to great complexity of xenobiotic transformation pathways in the living organisms, evaluation and optimization of metabolic stability remains a big challenge.

**Results:**

Here, we present a novel methodology for the evaluation and analysis of structural features influencing metabolic stability. To this end, we use a well-established explainability method called SHAP. We built several predictive models and analyse their predictions with the SHAP values to reveal how particular compound substructures influence the model’s prediction. The method can be widely applied by users thanks to the web service, which accompanies the article. It allows a detailed analysis of SHAP values obtained for compounds from the ChEMBL database, as well as their determination and analysis for any compound submitted by a user. Moreover, the service enables manual analysis of the possible structural modifications via the provision of analogous analysis for the most similar compound from the ChEMBL dataset.

**Conclusions:**

To our knowledge, this is the first attempt to employ SHAP to reveal which substructural features are utilized by machine learning models when evaluating compound metabolic stability. The accompanying web service for metabolic stability evaluation can be of great help for medicinal chemists. Its significant usefulness is related not only to the possibility of assessing compound stability, but also to the provision of information about substructures influencing this parameter. It can assist in the design of new ligands with improved metabolic stability, helping in the detection of privileged and unfavourable chemical moieties during stability optimization. The tool is available at https://metstab-shap.matinf.uj.edu.pl/.

## Background

It is not a mystery that the process of drug design and development is very complex and absorbs a huge amount of time and money [1, 2]. Although nowadays it significantly differs from the drug design strategies from the past (the emergence of new medicines used to be rather a result of serendipity and fortunate accidents [3]), it is still a subject to relatively high risk of failure. Nevertheless, the current strategies of searching for new drugs are much more structured and several steps can be distinguished within them, such as target identification, finding the lead structure, its optimization, preclinical studies and 3 phases of clinical tests [4, 5].

Finding a new active compound towards a particular target is just the first step in the long path of its possible transformation into a drug. Meeting the affinity requirements is not sufficient, as a compound needs to possess favourable physicochemical and pharmacokinetic properties as well, and it should not display any toxic effects [6–8]. Within the set of considered parameters it is also important to put attention to metabolic stability, because if a compound is transformed in the organism too quickly, it does not have enough time to induce a desired biological response [9].

Metabolic stability is one of the most difficult parameters to be predicted by computational tools due to extreme complexity of processes related to xenobiotic transformations in the living organisms. The main role in xenobiotic metabolism is played by cytochrome P450—a group of haemoprotein enzymes with monooxidase activity. Almost sixty CYP isoforms occur in human organisms; however, it is CYP3A4 that is responsible for metabolism of the majority of drugs [10–12].

A high number of processes that contribute to metabolic stability makes the correct prediction of this parameter a challenging task. As a result, publications on in silico tools for evaluating the speed of compound metabolism are scarce. Here, we mention a few examples of such studies. Schwaighofer et al. [13] analyzed compounds examined by the Bayer Schering Pharma in terms of the percentage of compound remaining after incubation with liver microsomes for 30 min. The human, mouse, and rat datasets were used with approximately 1000–2200 datapoints each. The compounds were represented by molecular descriptors generated with Dragon software and both classification and regression probabilistic models were developed with the AUC on the test set ranging from 0.690 to 0.835. Lee et al. [14] used MOE descriptors, E-State descriptors, ADME keys, and ECFP6 fingerprints to prepare Random Forest and Naïve Bayes predictive models for evaluation of compound apparent intrinsic clearance with the most effective method reaching 75% accuracy on the validation set. Bayesian approach was also used by Hu et al. [15] with accuracy of compound assignment to the stable or unstable class ranging from 75 to 78%. Jensen et al. [16] focused on more structurally consistent group of ligands (calcitriol analogues) and developed predictive model based on the Partial Least-Squares (PLS) regression, which was found to be 85% effective in the stable/unstable class assignment. On the other hand, Stratton et al. [17] focused on the antitubercular agents and applied Bayesian models to optimize metabolic stability of one of the thienopyrimidine derivatives. Arylpiperazine core was deeply examined in terms of in silico evaluation of metabolic stability by Ulenberg et al. [18] (Dragon descriptors and Support Vector Machines (SVM) were used) who obtained performance of R^2^ = 0.844 and MSE = 0.005 on the test set. QSPR models on a diverse compound sets were constructed by Shen et al. [19] with R^2^ ranging from 0.5 to 0.6 in cross-validation experiments and stable/unstable classification with 85% accuracy on the test set.

In silico evaluation of particular compound property constitutes great support of the drug design campaigns. However, providing explanation of predictive model answers and obtaining guidance on the most advantageous compound modifications is even more helpful. Searching for such structural-activity and structural-property relationships is a subject of Quantitative Structural-Activity Relationship (QSAR) and Quantitative Structural-Property Relationship (QSPR) studies. Interpretation of such models can be performed e.g. via the application of Multiple Linear Regression (MLR) or PLS approaches [20, 21]. Descriptors importance can also be relatively easily derived from tree models [20, 21]. Recently, researchers' attention is also attracted by the deep neural nets (DNNs) [21] and various visualization methods, such as the ‘SAR Matrix’ technique developed by Gupta-Ostermann and Bajorath [22]. The ‘SAR Matrix’ is based on the matched molecular pair (MMP) formalism, which is also widely used for QSAR/QSPR models interpretation [23, 24]. The work of Sasahara et al. [25] is one of the most recent examples of the development of interpretable models for studies on metabolic stability.

In our study, we focus on the ligand-based approach to metabolic stability prediction. We use datasets of compounds for which the half-lifetime (T1/2) was determined in human- and rat-based in vitro experiments. After compound representation by two key-based fingerprints, namely MACCS keys fingerprint (MACCSFP) [26] and Klekota & Roth Fingerprint (KRFP) [27], we develop classification and regression models (separately for human and rat data) with the use of three machine learning (ML) approaches: Naïve Bayes classifiers [28], trees [29–31], and SVM [32]. Finally, we use Shapley Additive exPlanations (SHAP) [33] to examine the influence of particular chemical substructures on the model’s outcome. It stays in line with the most recent recommendations for constructing explainable predictive models, as the knowledge they provide can relatively easily be transferred into medicinal chemistry projects and help in compound optimization towards its desired activity or physicochemical and pharmacokinetic profile [34]. SHAP assigns a value, that can be seen as importance, to each feature in the given prediction. These values are calculated for each prediction separately and do not cover a general information about the entire model. High absolute SHAP values indicate high importance, whereas values close to zero indicate low importance of a feature.

The results of the analysis performed with tools developed in the study can be examined in detail using the prepared web service, which is available at https://metstab-shap.matinf.uj.edu.pl/. Moreover, the service enables analysis of new compounds, submitted by the user, in terms of contribution of particular structural features to the outcome of half-lifetime predictions. It returns not only SHAP-based analysis for the submitted compound, but also presents analogous evaluation for the most similar compound from the ChEMBL [35] dataset. Thanks to all the above-mentioned functionalities, the service can be of great help for medicinal chemists when designing new ligands with improved metabolic stability. All datasets and scripts needed to reproduce the study are available at https://github.com/gmum/metstab-shap.

## Results

### Evaluation of the ML models

**Fig. 1:**
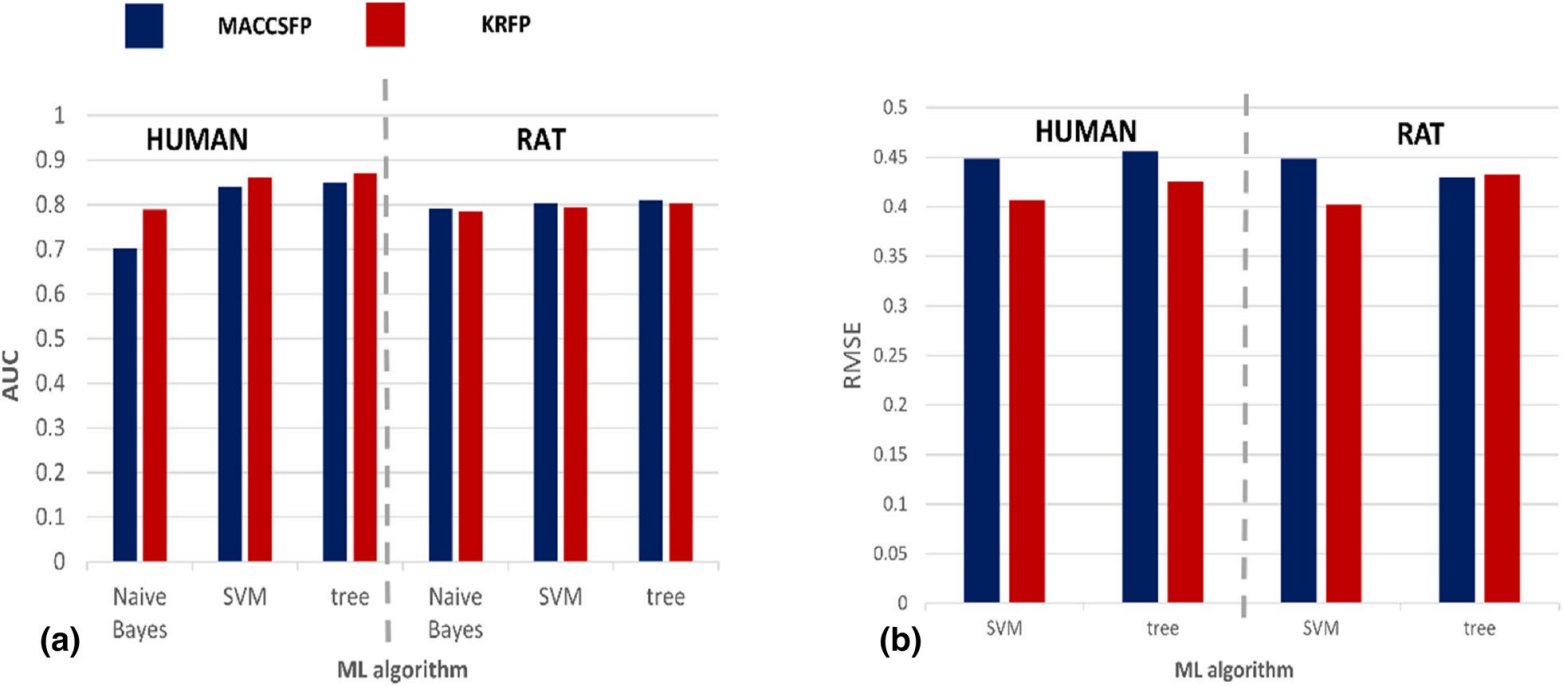
Global prediction power of the ML algorithms in **a** classification and **b** regression studies. The Figure presents global prediction accuracy expressed as AUC for classification studies and RMSE for regression experiments for MACCSFP and KRFP used for compound representation for human and rat data

We construct separate predictive models for two tasks: classification and regression. In the former case, the compounds are assigned to one of the metabolic stability classes (stable, unstable, and of middle stability) according to their half-lifetime (the T1/2 thresholds used for the assignment to particular stability class are provided in the Methods section), and the prediction power of ML models is evaluated with the Area Under the Receiver Operating Characteristic Curve (AUC) [36]. In the case of regression studies, we assess the prediction correctness with the use of the Root Mean Square Error (RMSE); however, during the hyperparameter optimization we optimize for the Mean Square Error (MSE). Analysis of the dataset division into the training and test set as the possible source of bias in the results is presented in the Appendix 1. The model evaluation is presented in Fig. 1, where the performance on the test set of a single model selected during the hyperparameter optimization is shown.

In general, the predictions of compound half-lifetimes are satisfactory with AUC values over 0.8 and RMSE below 0.4–0.45. These are slightly higher values than AUC reported by Schwaighofer et al. (0.690–0.835), although datasets used there were different and the model performances cannot be directly compared [13]. All class assignments performed on human data are more effective for KRFP with the improvement over MACCSFP ranging from ~ 0.02 for SVM and trees up to 0.09 for Naïve Bayes. Classification efficiency performed on rat data is more consistent for different compound representations with AUC variation of around 1 percentage point. Interestingly, in this case MACCSFP provides slightly more effective predictions than KRFP. When particular algorithms are considered, trees are slightly preferred over SVM (~ 0.01 of AUC), whereas predictions provided by the Naïve Bayes classifiers are worse**—**for human data up to 0.15 of AUC for MACCSFP. Differences for particular ML algorithms and compound representations are much lower for the assignment to metabolic stability class using rat data**—**maximum AUC variation is equal to 0.02.

When regression experiments are considered, the KRFP provides better half-lifetime predictions than MACCSFP for 3 out of 4 experimental setups**—**only for studies on rat data with the use of trees, the RMSE is higher by 0.01 for KRFP than for MACCSFP. There is ~ 0.02–0.03 RMSE difference between trees and SVMs with the slight preference (lower RMSE) for SVM. SVM-based evaluations are of similar prediction power for human and rat data, whereas for trees, there is ~ 0.03 RMSE difference between the prediction errors obtained for human and rat data.

### Regression vs. classification

Besides performing ‘standard’ classification and regression experiments, we also pose an additional research question related to the efficiency of the regression models in comparison to their classification counterparts. To this end, we prepare the following analysis: the outcome of a regression model is used to assign the stability class of a compound, applying the same thresholds as for the classification experiments. Accuracy of such classification is presented in Table 1.

**Table 1:** Comparison of accuracy of standard classification and class assignment based on the regression output

Dataset		Human		Rat	
Model	Representation	Class	Class. via regression	Class	Class. via regression
SVM	MACCS	**0.745**	0.695	0.676	**0.686**
	KRFP	**0.759**	0.672	0.676	**0.751**
Trees	MACCS	**0.737**	0.692	0.659	**0.686**
	KRFP	**0.734**	0.661	0.670	**0.676**

Comparison of efficiency of classification experiments (standard and using class assignment based on the regression output) expressed as accuracy. Higher values in a particular comparison setup are depicted in bold

Analysis of the classification experiments performed via regression-based predictions indicate that depending on the experimental setup, the predictive power of particular method varies to a relatively high extent. For the human dataset, the ‘standard classifiers’ always outperform class assignment based on the regression models, with accuracy difference ranging from ~ 0.045 (for trees/MACCSFP), up to ~ 0.09 (for SVM/KRFP). On the other hand, predicting exact half-lifetime value is more effective basis for class assignment when working on the rat dataset. The accuracy differences are much lower in this case (between ~ 0.01 and 0.02), with an exception of SVM/KRFP with difference of ~ 0.75. The accuracy values obtained in classification experiments for the human dataset are similar to accuracies reported by Lee et al. (75%) [14] and Hu et al. (75–78%) [15], though one must remember that the datasets used in these studies are different from ours and therefore a direct comparison is impossible.

### Global analysis of all ChEMBL data

**Fig. 2:**
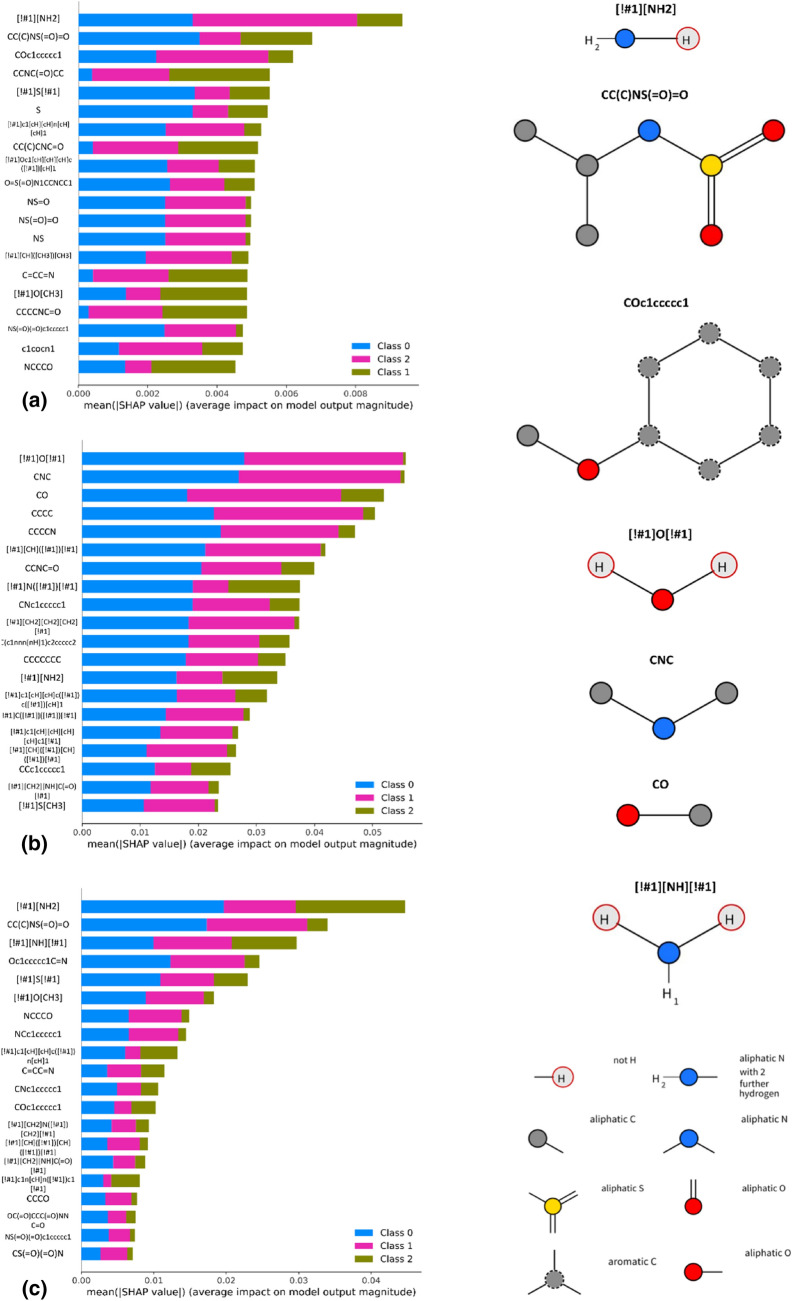
The 20 features which contribute the most to the outcome of classification models for **a** Naïve Bayes, **b** SVM, **c** trees constructed on human dataset with the use of KRFP

**Fig. 3:**
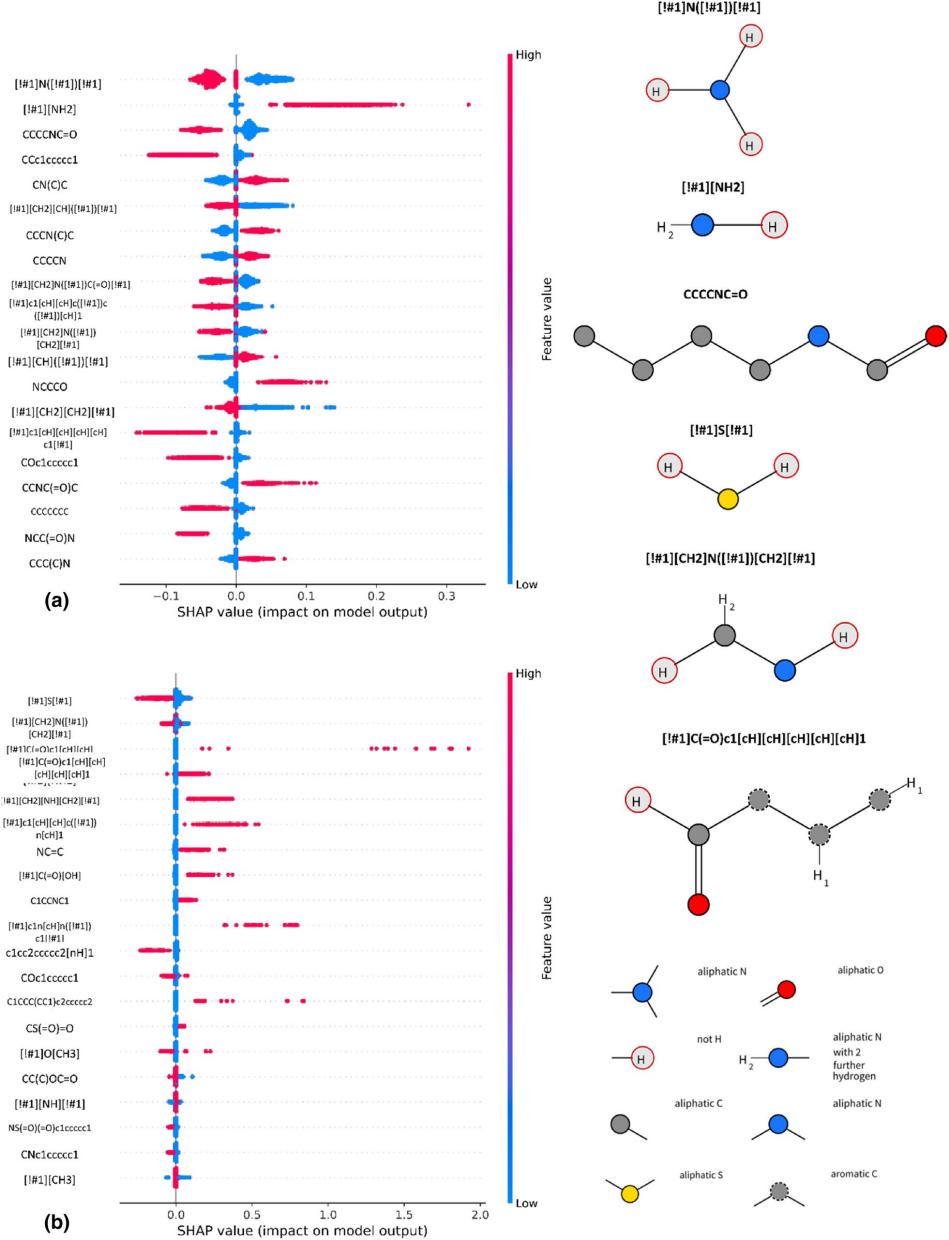
The 20 features which contribute the most to the outcome of regression models for **a** SVM, **b** trees constructed on human dataset with the use of KRFP

We analyzed the predictions obtained on the ChEMBL data with the use of SHAP values in order to find these substructural features, which have the highest contribution to particular class assignment (Fig. 2) or prediction of exact half-lifetime value (Fig. 3); class 0—unstable compounds, class 1—compounds of middle stability, class 2—stable compounds.

Analysis of Fig. 2 reveals that among the 20 features which are indicated by SHAP values as the most important overall, most features contribute rather to the assignment of a compound to the group of unstable molecules than to the stable ones**—**bars referring to class 0 (unstable compounds, blue) are significantly longer than green bars indicating influence on classifying compound as stable (for SVM and trees). However, we stress that these are averaged tendencies for the whole dataset and that they consider absolute values of SHAP. Observations for individual compounds might be significantly different and the set of highest contributing features can vary to high extent when shifting between particular compounds. Moreover, the high absolute values of SHAP in the case of the unstable class can be caused by two factors: (a) a particular feature makes the compound unstable and therefore it is assigned to this class, (b) a particular feature makes compound stable**—**in such case, the probability of compound assignment to the unstable class is significantly lower resulting in negative SHAP value of high magnitude.

For both Naïve Bayes classifier as well as trees it is visible that the primary amine group has the highest impact on the compound stability. As a matter of fact, the primary amine group is the only feature which is indicated by trees as contributing mostly to compound instability. However, according to the above-mentioned remark, it suggests that this feature is important for unstable class, but because of the nature of the analysis it is unclear whether it increases or decreases the possibility of particular class assignment.

Amines are also indicated as important for evaluation of metabolic stability for regression models, for both SVM and trees. Furthermore, regression models indicate a number of nitrogen- and oxygen-containing moieties as important for prediction of compound half-lifetime (Fig. 3). However, the contribution of particular substructures should be analyzed separately for each compound in order to verify the exact nature of their contribution.

**Fig. 4:**
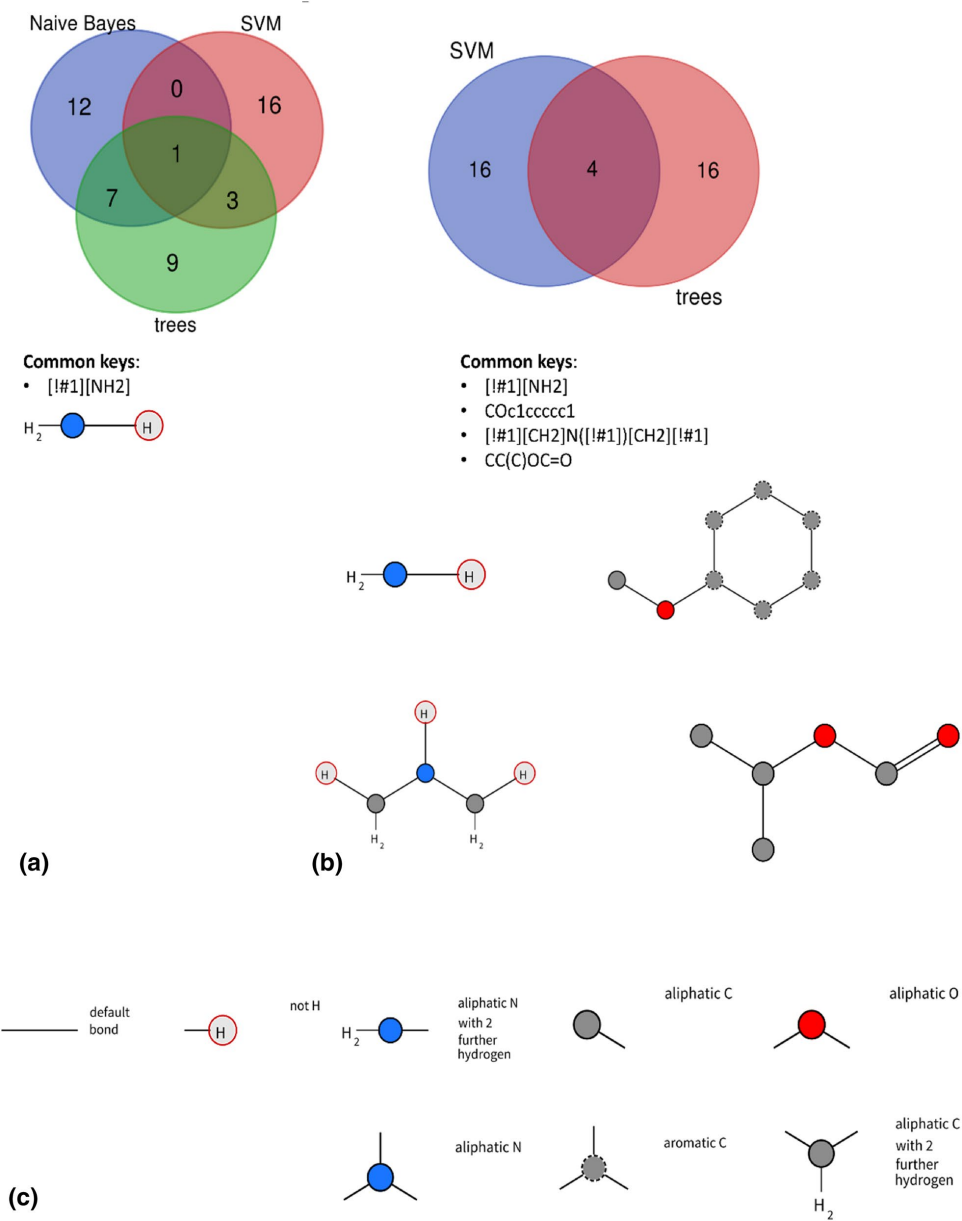
Overlap of important keys for **a** classification studies and **b** regression studies; c) legend for SMARTS visualization. Analysis of the overlap of the most important keys (in the number of 20) indicated by SHAP values for **a** classification studies and **b** regression studies; **c** legend for SMARTS visualization (generated with the use of SMARTS plus (https://smarts.plus/); Venn diagrams generated by http://bioinformatics.psb.ugent.be/webtools/Venn/

In order to examine to what extent the choice of the ML model influences the features indicated as important in particular experiment, Venn diagrams visualizing overlap between sets of features indicated by SHAP values are prepared and shown in Fig. 4. In each case, 20 most important features are considered.

When different classifiers are analyzed, there is only one common feature which is indicated by SHAP for all three models: the primary amine group. The lowest overlap between pairs of models occurs for Naïve Bayes and SVM (only one feature), whereas the highest (8 features) for Naïve Bayes and trees. For SVM and trees, the SHAP values indicate 4 common features as the highest contributors to the assignment to particular stability class. Nevertheless, we should remember that for Naïve Bayes the prediction accuracy was significantly lower than for SVM or trees; and therefore, the features indicated by this approach are also less reliable.

Finally, 4 features are common for SVM and trees in the case of regression experiments: the already mentioned primary amine group, alkoxy-substituted phenyl, secondary amine, and ester. This is in line with the intuition on the possible transformations that can occur for compounds containing these chemical moieties.

### Case studies

**Fig. 5:**
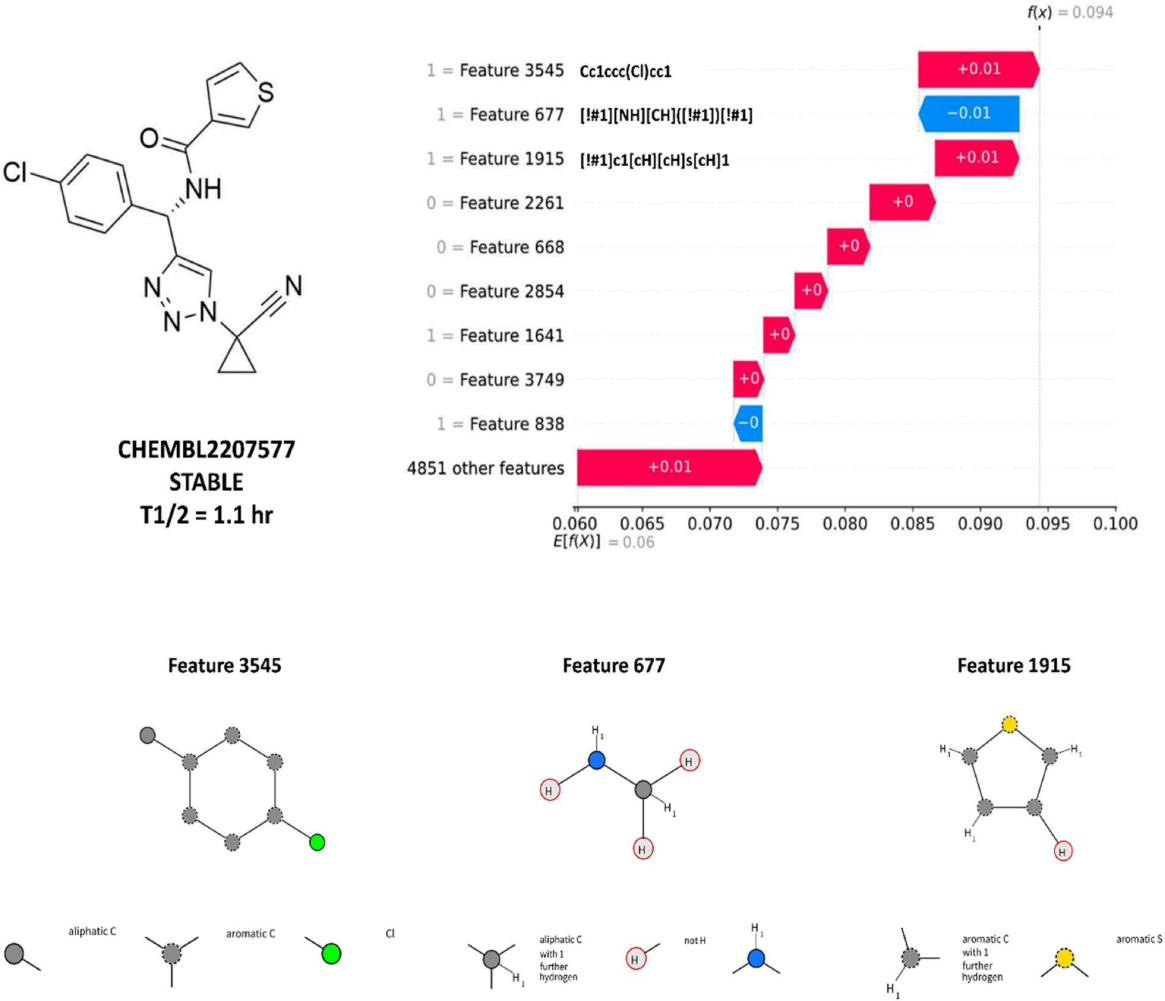
Analysis of the metabolic stability prediction for CHEMBL2207577 for human/KRFP/trees predictive model. Analysis of the metabolic stability prediction for CHEMBL2207577 with the use of SHAP values for human/KRFP/trees predictive model with indication of features influencing its assignment to the class of stable compounds; the SMARTS visualization was generated with the use of SMARTS plus (https://smarts.plus/)

In order to verify the applicability of the developed methodology on particular case, we analyze the output of an example compound (Fig. 5).

The highest contribution to the stability of CHEMBL2207577 is indicated to be the aromatic ring with the chlorine atom attached (feature 3545) and thiophen (feature 1915), the secondary amine (feature 677) lowers the probability of assignment to the stable class. All these features are present in the examined compounds and their metabolic stability indications are already known by chemists and they are in line with the results of the SHAP analysis.

### Web service

**Fig. 6:**
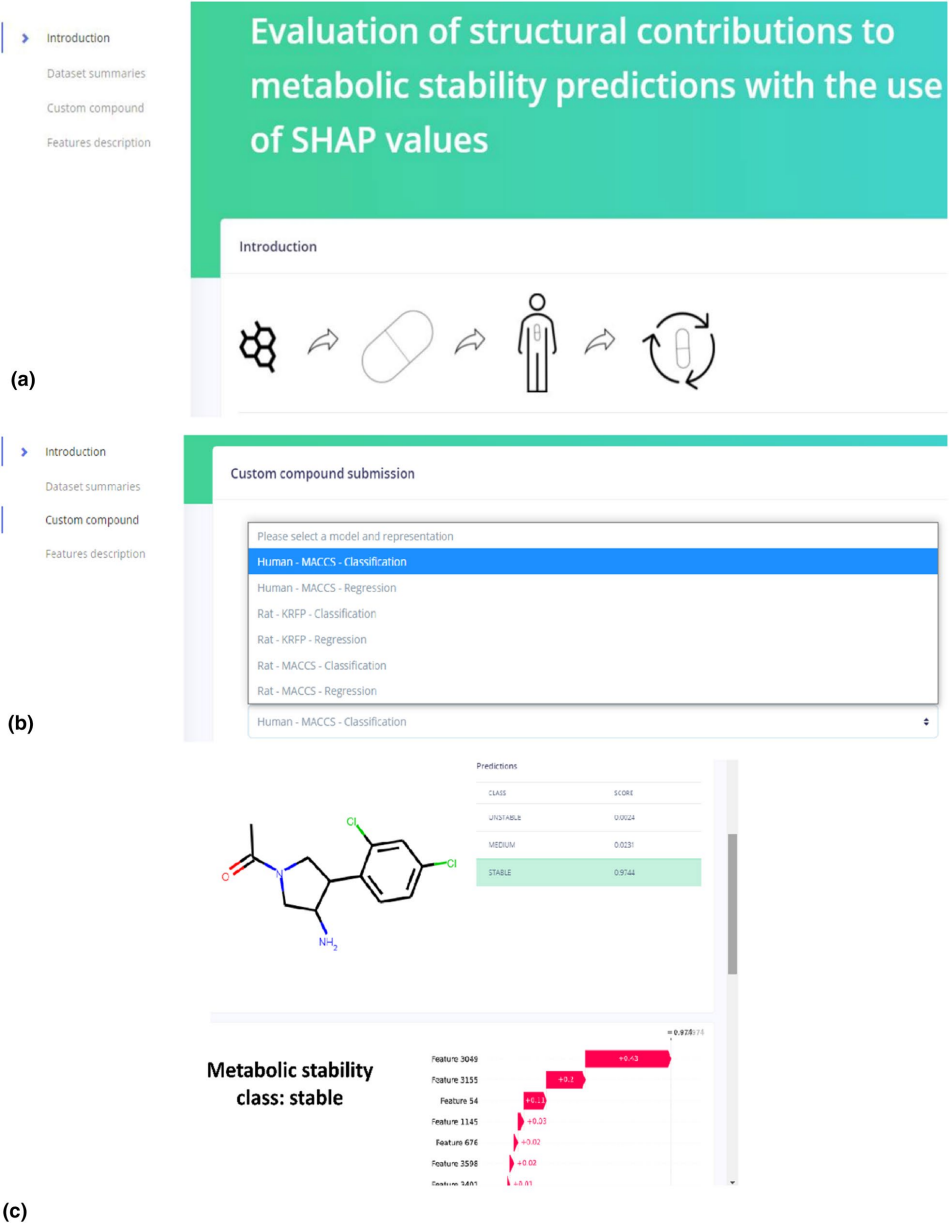
Screens of the web service **a** main page, **b** submission of custom compound, **c** stability predictions and SHAP-based analysis for a submitted compound. Screens of the web service for the compound analysis using SHAP values. **a** main page, **b** submission of custom compound for evaluation, **c** stability predictions for a submitted compound and SHAP-based analysis of its structural features

The results of all experiments can be analyzed in detail with the use of the web service, which can be found at https://metstab-shap.matinf.uj.edu.pl/. In addition, the user can submit their own compound and its metabolic stability will be evaluated with the use of the constructed models and the contribution of particular structural features will be evaluated with the use of the SHAP values (Fig. 6). Moreover, in order to enable manual comparisons, the most similar compound from the ChEMBL set (in terms of the Tanimoto coefficient calculated on Morgan fingerprints) is provided for each submitted compound (if the similarity is above the 0.3 threshold).

Obtaining such information enables optimization of metabolic stability as the substructures influencing this parameter are detected. Moreover, the comparison of several ML models and compound representations allows to provide a comprehensive overview of the problem.

**Fig. 7:**
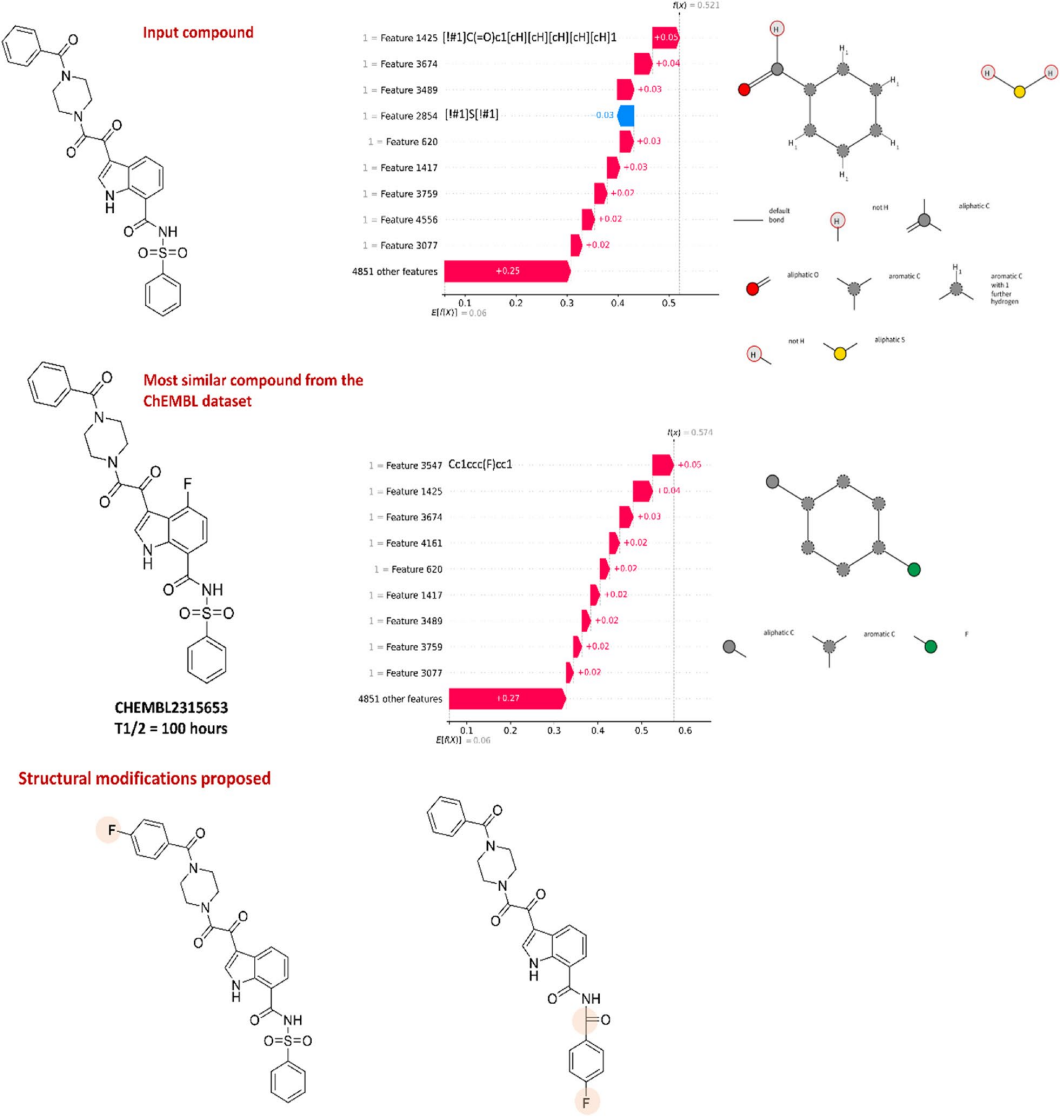
Custom compound analysis with the use of the prepared web service and output application to optimization of compound structure. Custom compound analysis with the use of the prepared web service, together with the application of its output to the optimization of compound structure in terms of its metabolic stability (human KRFP classification model was used); the SMARTS visualization generated with the use of SMARTS plus (https://smarts.plus/)

An example analysis of the output of the presented web service and its application in the compound optimization in terms of its metabolic stability is presented in Fig. 7.

The analysis of the submitted compound (evaluated in the classification studies as stable) indicates that the highest positive contribution to its metabolic stability has benzaldehyde moiety, and the feature which has a negative contribution to the assignment to the stable class is aliphatic sulphur. The most similar compound from the ChEMBL dataset is CHEMBL2315653, which differs from the submitted compound only by the presence of a fluorine atom. For this compound, the substructure indicated as the one with the highest positive contribution to compound stability is fluorophenyl. Therefore, the proposed structural modifications of the submitted compound involves the addition of the fluorine atom to the phenyl ring and the substitution of sulfone by ketone.

## Conclusions

In the study, we focus on an important chemical property considered by medicinal chemists**—**metabolic stability. We construct predictive models of both classification and regression type, which can be used for computational assessment of this parameter with the use of the provided on-line tool. Moreover, we use an explainability method called SHAP to develop a methodology for indication of structural contributors, which have the strongest influence on the particular model output. Finally, we prepared a web service, where user can analyze in detail predictions for CHEMBL data, or submit own compounds for metabolic stability evaluation. As an output, not only the result of metabolic stability assessment is returned, but also the SHAP-based analysis of the structural contributions to the provided outcome is given. In addition, a summary of the metabolic stability (together with SHAP analysis) of the most similar compound from the ChEMBL dataset is provided. All this information enables the user to optimize the submitted compound in such a way that its metabolic stability is improved. The web service is available at https://metstab-shap.matinf.uj.edu.pl/.

## Methods

### Data

We use CHEMBL-derived datasets describing human and rat metabolic stability (database version used: 23). We only use these measurements which are given in hours and refer to half-lifetime (T1/2), and which are described as examined on’Liver’,’Liver microsome’ or’Liver microsomes’. The half-lifetime values are log-scaled due to long tail distribution of the metabolic stability measurements. In case of multiple measurements for a single compound, we use their median value. In total, the human dataset comprises 3578 measurements for 3498 compounds and the rat dataset 1819 measurements for 1795 compounds. The resulting datasets are randomly split into training and test data, with the test set being 10% of the whole data set. The detailed number of measurements and compounds in each subset is listed in Table 2. Finally, the training data is split into five cross-validation folds which are later used to choose the optimal hyperparameters.

**Table 2:** Number of measurements and compounds in the ChEMBL datasets

Dataset	Subset	Number of measurements	Number of compounds
Human	Train	3221	3149
	Test	357	349
	Total	3578	3498
Rat	Train	1634	1616
	Test	185	179
	Total	1819	1795

The table presents the number of measurements and compounds present in particular datasets used in the study**—**human and rat data, divided into training and test sets

In our experiments, we use two compound representations: MACCSFP [26] calculated with the RDKit package [37] and Klekota & Roth FingerPrint (KRFP) [27] calculated using PaDELPy (available at https://github.com/ECRL/PaDELPy)—a python wrapper for PaDEL descriptors [38]. These compound representations are based on the widely known sets of structural keys—MACCS, developed and optimized by MDL for similarity-based comparisons, and KRFP, prepared upon examination of the 24 cell-based phenotypic assays to identify substructures which are preferred for biological activity and which enable differentiation between active and inactive compounds. Complete list of keys is available at https://metstab-shap.matinf.uj.edu.pl/features-description. Data preprocessing is model-specific and is chosen during the hyperparameter search.

For compound similarity evaluation, we use Morgan fingerprint, calculated with the RDKit package with 1024-bit length and other settings set to default.

### Tasks

We perform both direct metabolic stability prediction (expressed as half-lifetime) with regression models and classification of molecules into three stability classes (unstable, medium, and stable). The true class for each molecule is determined based on its half-lifetime expressed in hours. We follow the cut-offs from Podlewska et al. [39]:

-  ≤ 0.6**—**low stability,
- (0.6 − 2.32 > **—**medium stability,
-  > 2.32**—**high stability.

### Models

In our experiments, we examine Naïve Bayes classifiers, Support Vector Machines (SVMs), and several models based on trees. We use the implementations provided in the scikit-learn package [40]. The optimal hyperparameters for these models and model-specific data preprocessing is determined using five-fold cross-validation and a genetic algorithm implemented in TPOT [41]. The hyperparameter search is run on 5 cores in parallel and we allow it to last for 24 h. To determine the optimal set of hyperparameters, the regression models are evaluated using (negative) mean square error, and the classifiers using one-versus-one area under ROC curve (AUC), which is the average AUC of all possible pairwise combinations of classes. We use the scikit-learn implementation of ROC_AUC score with parameter multiclass set to ‘ovo’.

The hyperparameters accepted by the models and their values considered during hyperparameter optimization are listed in Tables 3, 4, 5, 6, 7, 8, 9. After the optimal hyperparameter configuration is determined, the model is retrained on the whole training set and evaluated on the test set.

**Table 3:** Hyperparameters accepted by different Naïve Bayes classifiers

	alpha	Fit_prior	norm	var_smoothing
BernoulliNB	✓	✓		
ComplementNB	✓	✓	✓	
GaussianNB				✓
MultinomialNB	✓	✓		

The table lists the hyperparameters which are accepted by different Naïve Bayes classifiers

**Table 4:** The values considered for hyperparameters for Naïve Bayes classifiers

Hyperparameter	Considered values
Alpha	0.001, 0.01, 0.1, 1, 10, 100
var_smoothing	1e−11, 1e−10, 1e−9, 1e−8, 1e−7, 1e−6, 1e−5, 1e−4
fit_prior	True, False
Norm	True, False

The table lists the values of hyperparameters which were considered during optimization process of different Naïve Bayes classifiers

**Table 5:** Hyperparameters accepted by different tree models

	n_estimators	max_depth	max_samples	splitter	max_features	bootstrap
ExtraTrees	✓	✓	✓			
DecisionTree		✓		✓	✓	
RandomForest	✓	✓	✓			✓

The table lists the hyperparameters which are accepted by different tree classifiers

**Table 6:** The values considered for hyperparameters for different tree models

Hyperparameter	Considered values
n_estimators	10, 50, 100, 500, 1000
max_depth	1, 2, 3, 4, 5, 6, 7, 8, 9, 10, 15, 20, 25, None
max_samples	0.5, 0.7, 0.9, None
splitter	Best, random
max_features	np.arrange(0.05, 1.01, 0.05)
bootstrap	True, False

The table lists the values of hyperparameters which were considered during optimization process of different tree models

**Table 7:** Hyperparameters accepted by SVMs with different kernels for classification experiments

kernel	c	loss	dual	penalty	gamma	coeff0	degree	tol	epsilon	Max_oter	probability
linear	✓	✓	✓	✓				✓			
rbf	✓				✓			✓		✓	✓
poly	✓				✓	✓	✓	✓		✓	✓
sigmoid	✓				✓	✓		✓		✓	✓

The table lists the hyperparameters which are accepted by different SVMs in classification experiments

**Table 8:** Hyperparameters accepted by SVMs with different kernels for regression experiments

kernel	c	loss	dual	penalty	gamma	Coeff0	degree	tol	epsilon	Max_oter	probability
linear	✓	✓	✓					✓	✓		
rbf	✓				✓			✓	✓	✓	
poly	✓				✓	✓	✓	✓	✓	✓	
sigmoid	✓				✓	✓		✓	✓	✓	

The table lists the hyperparameters which are by different SVMs in regression experiments

**Table 9:** The values considered for hyperparameters for different SVM models

hyperparameter	Considered values
C	0.0001, 0.001, 0.01, 0.1, 0.5, 1.0, 5.0, 10.0, 15.0, 20.0, 25.0
loss (SVC)	hinge, squared_hinge
loss (SVR)	epsilon_insensitive, squared_epsilon_insensitive
dual	True, False
penalty	11, 12
gamma	[auto, scale] + [10 ** i for i in range (− 6, 0)]
coef0	[10 ** i for i in range (− 6, 0)] + [0.0] + [10 ** i for i in range (− 1, − 7, − 1)]
degree	1…9
tol	1e−05, 0.0001, 0.001, 0.01, 0.1
epsilon	0.0001, 0.001, 0.01, 0.1, 1.0
max_iter	2000
probability	True

The table lists the values of hyperparameters which were considered during optimization process of different SVM models during classification and regression

### Explainability

We assume that if a model is capable of predicting metabolic stability well, then the features it uses might be relevant in determining the true metabolic stability. In other words, we analyse machine learning models to shed light on the underlying factors that influence metabolic stability. To this end, we use the SHapley Additive exPlanations (SHAP) [33]. SHAP allows to attribute a single value (the so-called SHAP value) for each feature of the input for each prediction. It can be interpreted as a feature importance and reflects the feature’s influence on the prediction. SHAP values are calculated for each prediction separately (as a result, they explain a single prediction, not the entire model) and sum to the difference between the model’s average prediction and its actual prediction. In case of multiple outputs, as is the case with classifiers, each output is explained individually. High positive or negative SHAP values suggest that a feature is important, with positive values indicating that the feature increases the model’s output and negative values indicating the decrease in the model’s output. The values close to zero indicate features of low importance.

The SHAP method originates from the Shapley values from game theory. Its formulation guarantees three important properties to be satisfied: local accuracy, missingness and consistency. A SHAP value for a given feature is calculated by comparing output of the model when the information about the feature is present and when it is hidden. The exact formula requires collecting model’s predictions for all possible subsets of features that do and do not include the feature of interest. Each such term if then weighted by its own coefficient. The SHAP implementation by Lundberg et al. [33], which is used in this work, allows an efficient computation of approximate SHAP values.

In our case, the features correspond to presence or absence of chemical substructures encoded by MACCSFP or KRFP. In all our experiments, we use Kernel Explainer with background data of 25 samples and parameter link set to identity.

The SHAP values can be visualised in multiple ways. In the case of single predictions, it can be useful to exploit the fact that SHAP values reflect how single features influence the change of the model’s prediction from the mean to the actual prediction. To this end, 20 features with the highest mean absolute SHAP value are plotted side by side starting from the actual prediction and the most important feature at the top. The SHAP values of the remaining features are summed and plotted collectively at the bottom of the plot and ending at the model’s average prediction. In case of classification, this process is repeated for each of the model outputs resulting in three separate plots**—**one for each of the classes.

The SHAP values for multiple predictions can be averaged to discover general tendencies of the model. Initially, we filter out any predictions which are incorrect, because the features used to provide an incorrect answer are of little relevance. In case of classification, the class returned by the model must be equal to the true class for the prediction to be correct. In case of regression, we allow an error smaller or equal to 20% of the true value expressed in hours. Moreover, if both the true and the predicted values are greater than or equal to 7 h and 30 min, we also accept the prediction to be correct. In other words, we use the following condition: $\widehat{y}$ is correct if and only if (0.8y ≤ $\widehat{y}$  ≤ 1.2y) or (y ≥ 7.5 and $\widehat{y}$ ≥ 7.5), where y is the true half-lifetime expressed in hours, and $\widehat{y}$ is the predicted value converted to hours. After finding the set of correct predictions, we average their absolute SHAP values to establish which features are on average most important. In case of regression, each row in the figures corresponds to a single feature. We plot 20 most important features with the most important one at the top of the figure. Each dot represents a single correct prediction, its colour the value of the corresponding feature (blue**—**absence, red**—**presence), and the position on the x-axis is the SHAP value itself. In case of classification, we group the predictions according to their class and calculate their mean absolute SHAP values for each class separately. The magnitude of the resulting value is indicated in a bar plot. Again, the most important feature is at the top of each figure. This process is repeated for each output of the model**—**as a result, for each classifier three bar plots are generated.

### Hyperparameter details

The hyperparameter details are gathered in Tables 3, 4, 5, 6, 7, 8, 9: Table 3 and Table 4 refer to Naïve Bayes (NB), Table 5 and Table 6 to trees and Table 7, Table 8, and Table 9 to SVM.

### Description of the GitHub repository

All scripts are available at https://github.com/gmum/metstab-shap/. In folder ‘models’ there are scripts which can be used to train the models presented in our work and in folder ‘metstab_shap’, the implementation to reproduce the full results, which includes hyperparameter tuning and calculation of SHAP values. We encourage the use of the experiment tracking platform Neptune (https://neptune.ai/) for logging the results, however, it can be easily disabled. Both datasets, the data splits and all configuration files are present in the repository. The code can be run with the use of Conda environment, Docker container or Singularity container. The detailed instructions to run the code are present in the repository.

## Figures and Tables

**Fig. 8:**
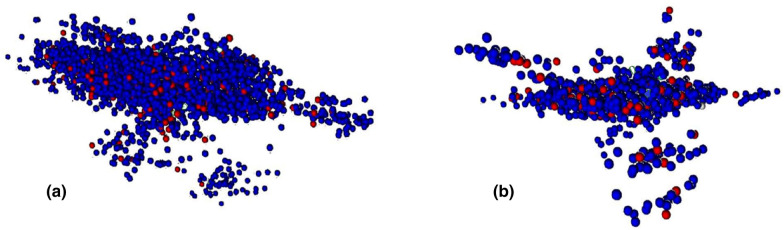
Chemical spaces of training (blue) and test set (red) for **a** human and **b** rat data. The figure presents visualization of chemical spaces of training and test set to indicate the possible bias of the results connected with the improper dataset division into the training and test set part. The analysis was generated using ECFP4 in the form of the principal component analysis with the webMolCS tool available at http://www.gdbtools.unibe.ch:8080/webMolCS/

**Fig. 9:**
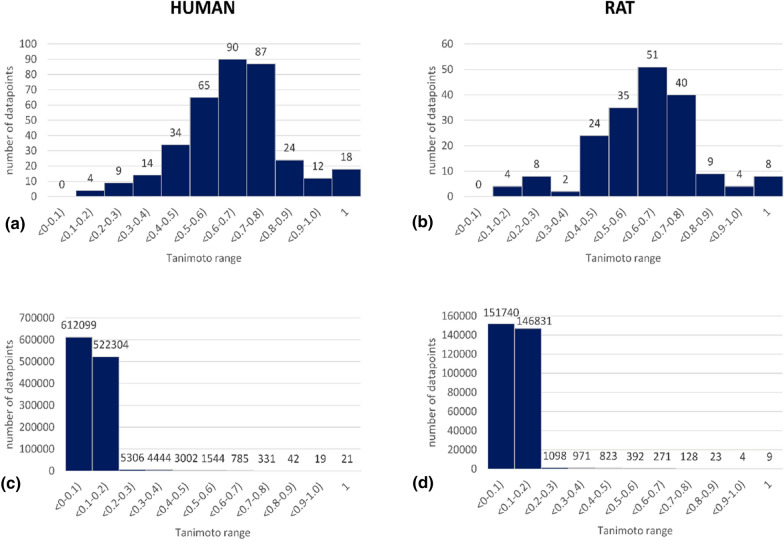
Tanimoto coefficients between training and test set for **a**, **b** the closest neighbour, **c**, **d** all training and test set representatives. The figure presents histograms of Tanimoto coefficients calculated between each representative of the training set and each element from the test set. **a**, **b** report only the highest values calculated for particular element from the test set and **c**, **d** present outcome of all pairwise comparisons

**Fig. 10:**
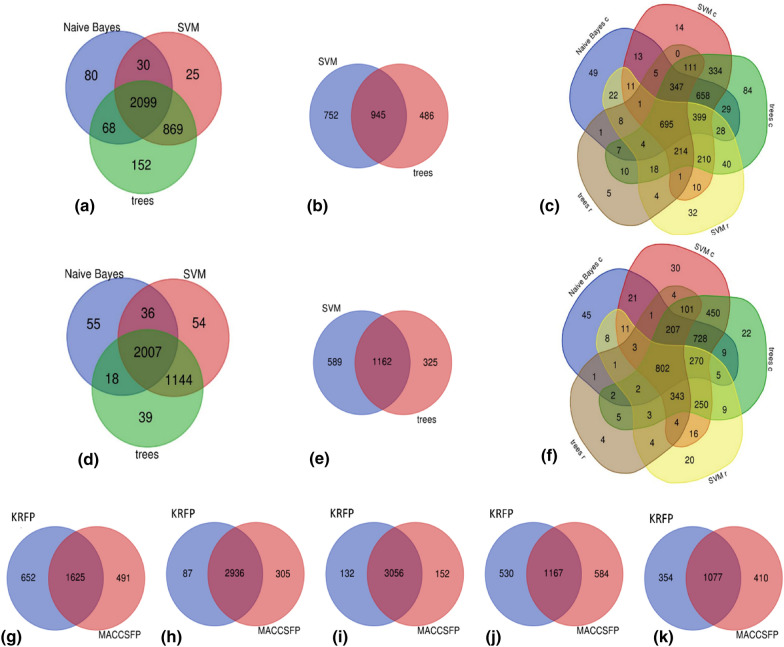
Venn diagrams for experiments on human data *presenting the number of correctly evaluated compounds in different setups (ML algorithms/compound representations)*: **a** classification on KRFP, **b** regression on KRFP, **c** classification and regression on KRFP, **d** classification on MACCSFP, **e** regression on MACCSFP, **f** classification and regression on MACCSFP, **g** classification with Naïve Bayes, **h** classification with SVM, **i** classification with trees, **j** regression with SVM, **k** regression with trees. The figure presents Venn diagrams showing the overlap between correctly predicted compounds in different experiments (different ML algorithms/compound representations) carried out on human data. Venn diagrams were generated with http://bioinformatics.psb.ugent.be/webtools/Venn/

**Fig. 11:**
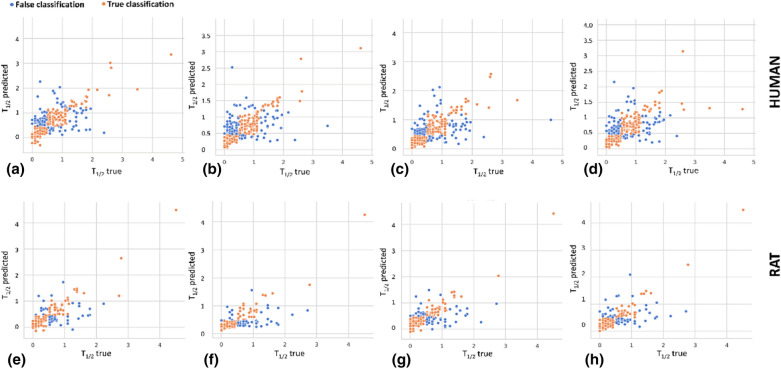
Parity plots showing the misclassification distribution in classification-via-regression experiments with reference to the half-lifetime values for **a** KRFP/SVM, **b** KRFP/trees, **c** MACCSFP/SVM, **d** MACCSFP/trees, **e** KRFP/SVM, **f** KRFP/trees, **g** MACCSFP/SVM, **h** MACCSFP/trees. The figure presents differences between true and predicted metabolic stability classes in the class assignment task performed based on the exact predicted value of half-lifetime in regression studies

**Fig. 12:**
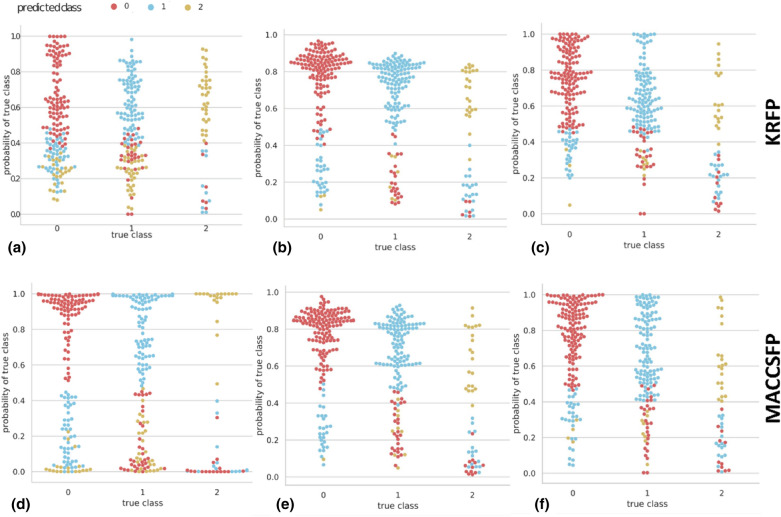
Analysis of the assignment correctness for models trained on human data: **a** NaïveBayes, **b** SVM, **c** trees, **d** NaïveBayes, **e** SVM, **f** trees. Class 0**—**unstable compounds, class 1**—**compounds of middle stability, class 2**—**stable compounds. The figure presents the distribution of probabilities of compound assignment to particular stability class, depending on the true class value for test sets derived from the human dataset. Each dot represent a single molecule, the position on x-axis indicates the correct class, the position on y-axis the probability of this class returned by the model, and the colour the class assignment based on model’s prediction

## Data Availability

Datasets and scripts enabling reproduction of all the results obtained in the study are available at https://github.com/gmum/metstab-shap. The online tool enabling the use of the developed methodology is available at https://metstab-shap.matinf.uj.edu.pl/.

## Appendix 1

### Training/test set analysis

In order to ensure that the predictions are not biased by the dataset division into training and test set, we prepared visualizations of chemical spaces of both training and test set (Fig. 8), as well as an analysis of the similarity coefficients which were calculated as Tanimoto similarity determined on Morgan fingerprints with 1024 bits (Fig. 9). In the latter case, we report two types of analysis**—**similarity of each test set representative to the closest neighbour from the training set, as well as similarity of each element of the test set to each element of the training set.

The PCA analysis presented in Fig. 8 clearly shows that the final train and test sets uniformly cover the chemical space and that the risk of bias related to the structural properties of compounds presented in either train or test set is minimized. Therefore, if a particular substructure is indicated as important by SHAP, it is caused by its true influence on metabolic stability, rather than overrepresentation in the training set.

The analysis of Tanimoto coefficients between training and test sets (Fig. 9) indicates that in each case the majority of compounds from the test set has the Tanimoto coefficient to the nearest neighbour from the training set in range of 0.6–0.7, which points to not very high structural similarity. The distribution of similarity coefficient is similar for human and rat data, and in each case there is only a small fraction of compounds with Tanimoto coefficient above 0.9. Next, the analysis of the all pairwise Tanimoto coefficients indicates that the overall similarity between training and test sets is low, with over 95% of Tanimoto values below 0.2.

## Appendix 2

### Prediction correctness analysis

In addition, the overlap of correctly predicted compounds for various models is examined to verify, whether shifting towards different compound representation or ML model can improve evaluation of metabolic stability (Fig. 10). The prediction correctness is examined using both the training and the test set. We use the whole dataset, as we would like to examine the reliability of the analysis carried out for all ChEMBL data in order to derive patterns of structural factors influencing metabolic stability.

In case of regression, we assume that the prediction is correct when it does not differ from the actual T1/2 value by more than 20% or when both the true and predicted values are above 7 h and 30 min.

The first observation coming from Fig. 10 is that the overlap of correctly classified compounds is much higher for classification than for regression studies. The number of compounds which are correctly classified by all three models is slightly higher for KRFP than for MACCSFP, although the difference is not significant (less than 100 compounds, which constitutes around 3% of the whole dataset).

On the other hand, the rate of correctly predicted compounds overlap is much lower for regression studies and MACCSFP seems to be more effective representation when the consensus for different predictive models is taken into account. Moreover, the total number of correctly evaluated compounds is also much lower for regression studies in comparison to standard classification (this is also reflected by the lower efficiency of classification via regression for the human dataset).

When both regression and classification experiments are considered, only 20–25% of compounds are correctly predicted by all classification and regression models. The exact percentage of compounds depends on the compound representation and is higher for MACCSFP. There is no direct relationship between the prediction correctness and the compound structure representation or its half-lifetime value. Considering the model pairs, the highest overlap is provided by Naïve Bayes and trees in ‘standard’ classification mode.

Examination of the overlap between compound representations for various predictive models show that the highest overlap occurs for trees**—**over 85% of the total dataset is correctly classified by both models. On the other hand, the lowest overlap for different compound representations within the classification models occurs for Naïve Bayes; however, it is also the model for which there is the lowest total number of correctly predicted compounds (less than 75% of the whole dataset). When regression models are compared, the fraction of correctly predicted compounds is higher for SVM, although the number of compounds correctly predicted for both compound representations is similar for both SVM and trees (~ 1100, a slightly higher number for SVM).

Another type of prediction correctness analysis was performed for regression experiments with the use of the parity plots for ‘classification via regression’ experiments (Fig. 11).

Figure 11 indicates that there is no apparent correlation between the misclassification distribution and the half-lifetime values as the models misclassify molecules of both low and high stability.

Analogous analysis was performed for the classifiers (Fig. 12). One general observation is that in case of incorrect predictions the models are more likely to assign the compound to the neighbouring class, e.g. there is higher probability of the assignment of stable compounds (yellow dots) to the class of middle stability (blue) than to the unstable class (red). For compounds of middle stability, there is no direct tendency of class assignment when the prediction is incorrect**—**there is similar probability of predicting such compounds as stable and unstable ones. In the case of classifiers, the order of classes is irrelevant; therefore, it is highly probable that the models during training gained the ability to recognize reliable features and use them to correctly sort compounds according to their stability.

Evaluation of the predictive power of the obtained models allows us to state, that they are capable of assessing metabolic stability with high accuracy. This is important because we assume that if a model is capable of making correct predictions about the metabolic stability of a compound, then the structural features, which are used to produce such predictions, might be relevant for provision of desired metabolic stability. Therefore, the developed ML models underwent deeper examination to shed light on the structural factors that influence metabolic stability.
